# Do alternative resources dampen functional responses of native but not alien gammarids?

**DOI:** 10.1002/ece3.9262

**Published:** 2022-09-09

**Authors:** Ross N. Cuthbert, Syrmalenia G. Kotronaki, Jasmin C. Hütt, Elisabeth Renk, Niklas Warlo, Elizabeta Briski

**Affiliations:** ^1^ GEOMAR Helmholtz‐Zentrum für Ozeanforschung Kiel Kiel Germany; ^2^ School of Biological Sciences Queen's University Belfast Belfast UK

**Keywords:** Baltic Sea, functional response, *Gammarus locusta*, *Gammarus tigrinus*, *Pontogammarus maeoticus*, predator–prey interaction

## Abstract

While aquatic invasive predators are among the most impactful trophic groups, we lack the understanding of whether alternative food resources mediate adverse predatory effects and stabilize native prey communities. Here, we use comparative functional responses to examine the influence of alternative food resources (*Fucus* sp.) on predator–prey interaction strengths from three gammarid crustaceans, with one native (*Gammarus locusta*) and two existing and emerging invasive (*Gammarus tigrinus*, *Pontogammarus maeoticus*, respectively) species, towards larval chironomid prey. All gammarids exhibited Type II functional responses, irrespective of the presence of alternative seaweed disks. *Fucus* sp. disks significantly reduced predation rates overall; however, significant reductions in maximum feeding rates (i.e., functional response magnitudes) were only evident in the native species and not for the two invaders. Our results thus may suggest that alternative resources dampen the predatory interaction strength of native but not invasive alien species, concerning these three study organisms. This potentially exacerbates the impacts of invasive predators relative to natives in diverse communities. Studies should increasingly consider alternative resources when quantifying ecological impacts of current and future invasive alien species compared with natives.

## Graphical abstract

Alternative food resources may alter the predatory impact of invasive species compared to natives. We find that alternative seaweed food sources reduce the interaction strengths of predatory native gammarids, but not in invasive ones.

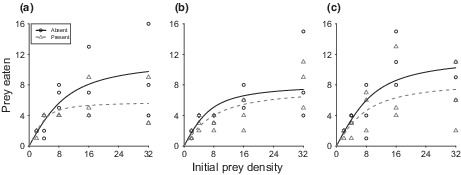

## 1 INTRODUCTION

Given their openness and interconnection through a broad range of transport pathways and vectors (e.g., shipping routes such as Suez Canal; Bailey et al., 2020), seas are especially vulnerable to biological invasions and their impacts (e.g., affecting abundance and behavior of native species; causing economic costs to sectors such as fisheries; Anton et al., 2019; Cuthbert et al., 2021). Recent works have identified predators to be among the most disruptive to marine ecosystems, although species displaying omnivory (i.e., consumption of multiple trophic groups) might be more innocuous in terms of impact (Anton et al., 2019). Accordingly, the level of impact of marine invaders might be altered by their feeding ecology and resource preferences, necessitating screening of potential ecological impacts under these contexts. On the one hand, omnivory could reduce the impact on any single trophic group due to resource switching, but on the other hand, it could permit broad‐ranging impacts by invasive consumers on multiple taxa (Anton et al., 2019; Oaten & Murdoch, 1975).

Gammarid crustaceans represent some of the most well‐studied and impactful invasive alien species, with invasion histories across a broad geographic range (Cuthbert et al., 2020; Grabowski et al., 2007; Paiva et al., 2018). Invasive gammarid species have considerable impacts (i.e., reductions in populations of native species) on native communities through the outcompeting of native species, intensification of trophic interactions, and disruption of biophysical structures (Grabowski et al., 2007). For example, the “killer shrimp” *Dikerogammarus villosus* impacts fish populations through predation of egg and larval stages of carp and trout, and to a greater extent than native gammarids (Taylor & Dunn, 2017). However, invasive gammarids have also been shown to exhibit a generalist dietary, whereby predation can be reduced in the presence of alternative food resources differentially between invaders and natives (Médoc et al., 2018). Alternative resources and their relative availability may thus potentially reduce the extent of ecological impacts on benthic prey communities. Nevertheless, emergent effects of alternative resources have been seldom assessed in gammarid invaders, particularly in brackish and marine seas—such as the Baltic Sea—which have been invaded at high rates (Casties et al., 2016; Cuthbert et al., 2020). If invasive alien species exhibit a greater animal prey preference than native species over alternative resource types, transitions from native to invasive populations might have cascading effects on lower trophic groups through intensified predator–prey relationships.

The functional response is one classical approach for quantifying interaction strengths between consumers and resources (Holling, 1959). As a means to measure resource use as a function of resource density, the functional response allows density dependence to be elucidated under a broad range of biotic and abiotic contexts. In turn, both form and type of functional responses enable implications for resource stability to be inferred (Dick et al., 2014; Hassell, 1978). Functional responses follow three common forms: linear Type I, hyperbolic Type II and sigmoid Type III. Impactful invasive alien species have been repeatedly characterized as having a higher magnitude functional response than trophically analogous native species (Dick et al., 2014), with a typically greater attack rate (i.e., slope of the feeding rate at low resource densities), shorter handling time (i.e., feeding rate asymptote at high resource densities) and thus greater maximum feeding rate (Cuthbert et al., 2019), coupled with higher field abundances (Dick et al., 2017). As the functional response allows for the consideration of context‐dependencies, it can be a useful tool to disentangle emergent effects of alternative resources for interaction strengths among invasive and native gammarids. For example, leaf litter provisioning has previously reduced the predatory feeding difference between native and invasive gammarids (Médoc et al., 2018). However, comparative functional responses have also been shown to differ among populations of invasive alien species (e.g., Boets et al., 2019).

Here, we therefore employ a comparative functional response approach to examine the predatory interaction strengths of a native gammarid in relation to two invasive alien species, with and without the presence of alternative seaweed resources. We compare the interactions of the native and locally dominant *Gammarus locusta* (Linnaeus 1758) in the Baltic Sea, associated with macroalgae (Andersson et al., 2009), to an established and widespread invasive alien species from North America, *Gammarus tigrinus* Sexton 1939, and an emerging invasive alien species, *Pontogammarus maeoticus* (Sovinskij 1894) from the Ponto‐Caspian region. Gammarid invasion success has been linked to several traits, such as brood size, fecundity, early maturation, and high generation numbers (Grabowski et al., 2007), and environmental tolerances (Paiva et al., 2018), and fecundity indices of *G. tigrinus* and *P. maeoticus* have been shown to be greater than resident native species (Dickey et al., 2020). The association and grazing of *G. tigrinus* on *Fucus* sp. and other seaweeds native to the Baltic has already been observed (Orav‐Kotta & Kotta, 2009), and habitats overlap with *G. locusta* and other native gammarid species (Kotta et al., 2011; Reisalu et al., 2016; Rewicz et al., 2019). Other prominent coastal gammarid species in the sampled area of the Baltic Sea include the native *Gammarus oceanicus* and *Gammarus salinus*. *Pontogammarus maeoticus* is not established in the Baltic Sea but is invasive elsewhere (Alexandrov et al., 2007; Özbek, 2011), and may be less familiar with algal resources from the Baltic Sea, and thus be more predatory or familiar with different resources in its native habitat. We hypothesize that (i) functional responses of the two invasive alien species tested here (*G. tigrinus* and *P. maeoticus*) will be significantly higher (i.e., greater attack and maximum feeding rates) than that of the native gammarid (*G. locusta*), but that (ii) alternative resource effects will differ between these tested invasive alien and native species, whereby predation rates are reduced in the native but not invasive alien species.

## 2 METHODS

### 2.1 Animal collection and maintenance

One native and two invasive gammarid species, *G. locusta*, *G. tigrinus*, and *P. maeoticus*, were obtained from laboratory cultures maintained for several years at GEOMAR Helmholtz Centre for Ocean Research Kiel. Although maintained in the laboratory for long periods of time, this length of acclimation enabled comparability among species given that one was not regionally available (i.e., *P. maeoticus*), although we acknowledge potential effects on behavior due to long‐term laboratory housing. Laboratory conditions were 18°C under a 12:12 light and dark photoperiod. Originally, *G. locusta* was obtained from Falkenstein Beach, Kiel, Germany; *G. tigrinus* from Travemünde, Lübeck, Germany; and *P. maeoticus* from the South Caspian Sea, near Jefrud, Iran. Sampling locations for all three species were shallow littoral sandy‐bottom substrates, with the *G. locusta* location characterized by brown algae *Fucus* sp. and blue mussels *Mytilus* sp., *G. tigrinus* with reeds *Phragmites* sp. and brown algae *Dictyosiphon* sp., while that of *P. maeoticus* was with green algae *Cladophora* sp. The salinities of the three locations range between 12–18, 4–12, and 4–11 ppt, respectively, with temperatures between 2–23, 2–24, and 10–28°C (E. Briski and A. Mirzajani, personal observations). In the laboratory, all three species were maintained in separate 56 L aquaria with an internal filtration system and a mixture of Baltic Sea water and dechlorinated tap water (5 μm‐filtered) to match the typical salinity of their collection site (*G. locusta*: 13 ppt; *G. tigrinus*: 10 ppt; *P. maeoticus*, 10 ppt). Gammarids were fed ad libitum bi‐weekly with a mixture of crushed crustacean food (Tetra Mix, Tetra Crusta, and Dr. Shrimp Healthy). Habitat in the form of sand, artificial weeds, and ceramic tubes were provided in aquaria to simulate natural structural complexities as far as possible. Brown algae (*Fucus* sp.), readily consumed by gammarids (R.N. Cuthbert personal observations), were collected from Falkenstein Beach (16 ppt salinity) and transported to the same laboratory 1 day before each trial. Live chironomid larval prey were purchased commercially for use in the functional response experiments (total length ± SD: 10.50 ± 1.56 mm) from ZOO and Co. Knutzen, Kiel and kept refrigerated until use.

### 2.2 Experimental procedure

We conducted a full‐factorial experiment to examine the influence of (i) an alternative algal resource (ii) gammarid predator species and (iii) chironomid prey density on predator–prey interaction strengths. Prior to each feeding experiment, predators of either sex were starved for 24 h in separate 5 L aquaria (25 × 15 × 15 cm) containing a 5 μm‐filtered 12 ppt salinity mixture of Baltic Sea‐dechlorinated tap water, with ceramic stones for habitat complexity (total length ± SD: *G. locusta*, 11.51 ± 1.36 mm; *G. tigrinus*, 10.87 ± 1.47 mm; *P. maeoticus*, 13.17 ± 1.19 mm). Each aquarium had continuous aeration and was kept at 18°C. The starvation process ensured the standardization of hunger levels among the focal species. The salinity regime (12 ppt) was selected as a reasonable mid‐point given that the gammarids were cultured in slightly different media prior to experimentation (i.e., 10–13 ppt). However, gammarids are frequently euryhaline (Cuthbert et al., 2020) and we did not observe any adverse transitional salinity effects on gammarids (i.e., increased mortality).

Feeding experiments were conducted in 1 L plastic jars (9 × 9 × 14 cm) containing 500 ml of 12 ppt salinity water. Each jar was allocated a predefined seaweed disk treatment (2 levels: absent or present, i.e., 0 or 10 apical disks of 8 mm diameter each, with the latter representing an overabundance as per pilot trials), predator species (4 levels: *G. locusta*, *G. tigrinus*, *P. maeoticus*, predator‐free control) and chironomid prey density (5 levels: 2, 4, 8, 16, 32 items), with each treatment group replicated three times and thus yielding 120 total experimental units (i.e., 2 × 4 × 5 × 3 = 120). We acknowledge the relatively small sample size per experimental group but found this sample size to be sufficient to fit functional response models (see later). A full set of replicates was run on three separate experimental occasions, with each treatment therein completely randomized to avoid positional effects. We did not reuse predators in any treatment to mitigate predator learning as far as possible. After the 24 h starvation period, gammarids were introduced into their respective treatment arenas (one gammarid per arena) and allowed to feed for 21 h. After this feeding period, gammarids were carefully removed using a wide‐ended pipette, and the remaining live and uneaten prey were quantified to enumerate prey consumption (i.e., numbers of prey killed and eaten).

### 2.3 Statistical analyses

Generalized linear models assuming a quasi‐binomial error distribution with logit links were used to examine feeding rates of gammarids according to seaweed treatment (2‐level factor), predator treatment (3‐level factor), and prey density (continuous term), and their two‐ and three‐way interactions. A quasi‐binomial family was used given significant evidence for overdispersion in the equivalent binomial model, indicated through statistical comparison of fitted and simulated residuals (DHARMa package: Hartig, 2021). *F*‐tests were computed through analysis of deviance (car package: Fox & Weisberg, 2019), with the final model including only significant terms via backward step deletion.

Logistic regression was used to infer functional response types, whereby a Type II response is evidenced by a significant negative first‐order term (Juliano, 2001). This was done separately for the six different seaweed and predator treatment combinations across prey densities. Because all treatments exhibited Type II functional responses, and we did not replace prey as they were consumed, we fit Rogers' random predator equation to each dataset (Rogers, 1972; frair package: Pritchard et al., 2017):

(1) $$N_{e}=N_{0}\left(1-\exp\left(a\left(N_{e}h-T\right)\right)\right)$$

where *N*_*e*_ is the number of prey eaten, *N*_0_ is the initial density of prey, *a* is the attack rate, *h* is the handling time (reciprocally the maximum feeding rate, 1/*h*) and *T* is the total experimental period. Because *N*_*e*_ appears on both sides of equation 1, the solution was found using Lambert's transcendental equation (Bolker, 2008). The difference (delta) method was then used to compare each functional response parameter (*a* and *h*) between seaweed treatments pairwise for individual predator species using indicator variables (Juliano, 2001; Pritchard et al., 2017). This was achieved by explicitly modeling the difference (delta) of each optimized functional response parameter between seaweed groups within species. All statistical analyses were computed in R v4.0.2 (R Core Team, 2020).

## 3 RESULTS

The vast majority of control prey remained intact (>98%) and therefore we did not adjust observed consumption rates in treatments with gammarids. No individual gammarid consumed all of the available seaweed, with the majority of disks remaining in all replicates. However, gammarids consumed significantly lower proportions of available chironomid prey in the presence of seaweed overall (*F*_1,87_ = 7.17, *p* = .01), with over 30% more prey consumed on average in the absence of seaweed compared with in its presence. Prey consumption rates also responded significantly negatively to initial prey densities across species and seaweed treatments (*F*_1,87_ = 75.55, *p* < .001). There was no significant difference in chironomid consumption rates among predators overall (*F*_2,87_ = 1.00, *p* = .37), or significant two‐ or three‐way interaction terms (all *p* > .05; Table 1).

**TABLE 1:** Quasi‐binomial generalized linear model results, considering gammarid feeding rates as a function of seaweed presence, predator species, and prey density. *F*‐values were returned via backward step deletion.

Term	*F*‐value	*p*‐Value
**Seaweed**	**7.17**	**.01**
Predator	1.00	.37
**Prey**	**75.55**	**<.001**
Seaweed:Predator	0.24	.79
Seaweed:Prey	0.06	.81
Predator:Prey	1.28	.28
Seaweed:Predator:Prey	0.45	.64

Significant terms are boldened

**FIGURE 1:**
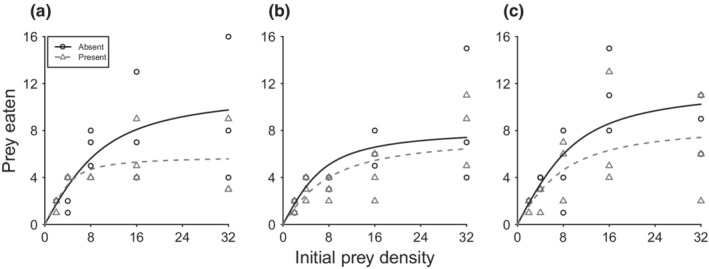
Functional responses towards chironomid prey in the absence (0 disks) and presence (10 disks) of *Fucus* sp. by (a) *Gammarus locusta*, (b) *Gammarus tigrinus*, and (c) *Pontogammarus maeoticus*. Lines represent the initial fits from Rogers' random predator equation, and points are the raw data per treatment.

**FIGURE 2:**
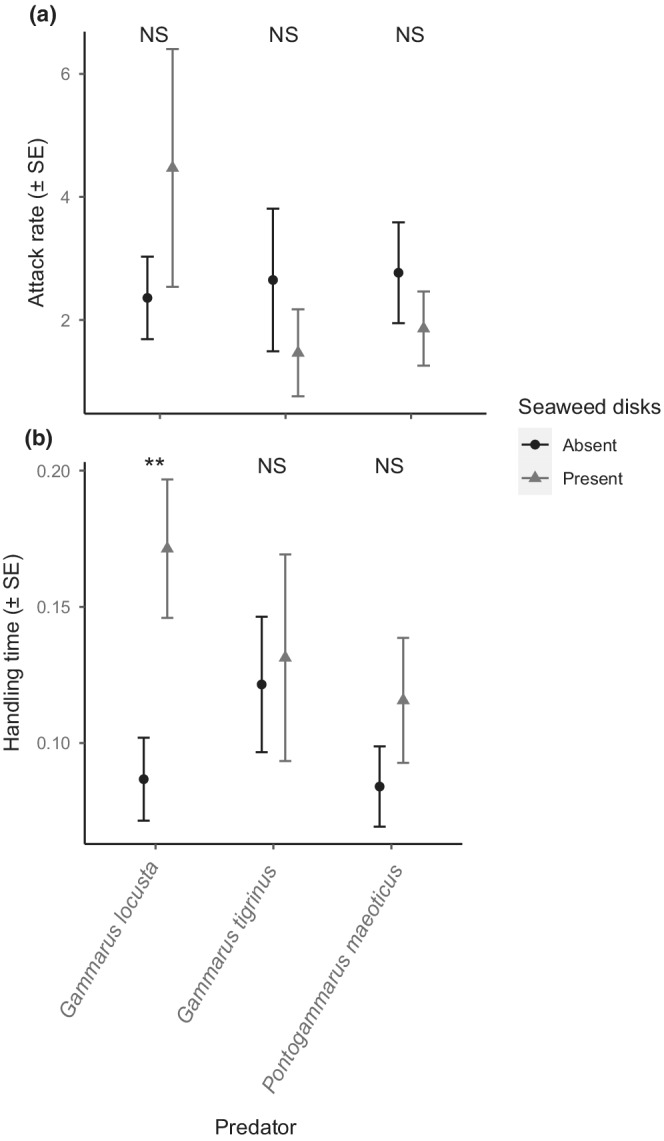
Functional response parameter estimates among gammarid species towards chironomid prey, considering (a) attack rates and (b) handling times, in the absence (0 disks) and presence (10 disks) of *Fucus* sp. Asterisks denote levels of statistical clarity [*p* < .05*; *p* < .01**; *p* < .001***; nonsignificant (NS)].

All gammarid species displayed significant Type II functional responses between seaweed treatments, with significant *a* and *h* estimates returned in all cases (Table 2; Figure 1). For *G. locusta*, the presence of seaweed resulted in a significant lengthening of handling times towards prey (z = 2.85, *p* = .004; Figure 2), with maximum feeding rates approximately halved in the presence of seaweed (Table 2). Attack rates of *G. locusta* were not significantly altered by the presence of seaweed (*z* = 1.033, *p* = .30). Neither attack rates nor handling times were significantly affected by the presence of seaweed in the case of *G. tigrinus* (*a*, z = 0.87, *p* = .38; *h*, *z* = 0.22, *p* = .83) and *P. maeoticus* (*a*, z = 0.89, *p* = .37; *h*, z = 1.16, *p* = .25). Accordingly, the effects of seaweed on handling times and, reciprocally, maximum feeding rates were only statistically clear in the case of native *G. locusta*, whereas the two invasive alien species exhibited conserved predator–prey interaction strengths between seaweed treatments.

**TABLE 2:** Functional response first‐order terms (logistic regression estimates), attack rates, handling times, and maximum feeding rates (random predator equation) according to seaweed and gammarid predator treatments.

Predator	Seaweed	First‐order term	Attack rate (*a*)	Handling time (*h*)	Max. feeding rate (1/*h*)
*Gammarus locusta*	Absent	−0.075***	2.359***	0.087***	11.494
	Present	−0.097***	4.471*	0.171***	5.848
*Gammarus tigrinus*	Absent	−0.063***	2.650*	0.122***	8.197
	Present	−0.043**	1.467*	0.131***	7.634
*Pontogammarus maeoticus*	Absent	−0.088***	2.768***	0.084***	11.905
	Present	−0.077***	1.861**	0.116***	8.621

Asterisks denote levels of statistical clarity (*p* < .05*; *p* < .01**; *p* < .001***, i.e., the difference of each parameter from zero

## 4 DISCUSSION

We demonstrated here, using comparative functional responses, that the predatory interaction strengths of native *G. locusta* but not *G. tigrinus* or *P. maeoticus* towards prey were significantly dampened by alternative food resources, using a study system comprising one native and two existing and emerging invasive alien gammarids. This thus confirms our second hypothesis (i.e., that predation by *G. locusta* is more dampened by alternative food resources than these two aliens) but rejects our first one (i.e., that the alien species show higher magnitude functional responses). These results suggest that, following invasion into communities with diverse resource types, these invasive alien species could be less affected by alternative resources, which might lead to destabilizing effects on some trophic groups. Equally, given the functional role of gammarids as shredders (Kelly et al., 2002), the invasion could reduce decomposition in aquatic ecosystems. In turn, our results suggest that considerations for diverse community structures should be increasingly made in impact assessments for invasive alien species. Whether these effects hold for other invader‐native comparator groups largely remains to be tested.

Despite significant lengthening of handling times in the native species, which led to reductions in maximum feeding rates in the presence of seaweed, the Type II model of the functional response was the most appropriate across treatment groups. This corroborates with other studies considering invasive and native gammarids, where Type II feeding responses have been evidenced (e.g., Cuthbert et al., 2018; Médoc et al., 2018). However, the addition of seaweed disks might have alternatively been expected to fundamentally alter the type of functional response, from a hyperbolic Type II to sigmoid Type III, characterized by emergent refuge effects for prey at low densities (Hassell, 1978). That is because consumers may exhibit a tendency to switch between resources depending on their relative availability within the environment, whereby rare resources are disproportionately avoided and abundant ones disproportionately targeted. However, a lack of switching propensity has already been evidenced in gammarids (Cuthbert et al., 2018), with consistent feeding preferences towards particular prey types displayed, irrespective of their environmental availability. Similarly, functional responses have been shown to not deviate from Type II forms in the presence of alternative resources considering freshwater gammarid invaders, but alternative resource inclusion induced greater predation similarities between invasive‐native comparators (Médoc et al., 2018). Considering the three gammarid species of the current study, native predator maximum feeding rates were reduced most in the presence of *Fucus* sp. disks. Nevertheless, the generalized linear model results on raw consumption rates did not corroborate a significant effect of seaweed among species in the functional responses, as the interaction effect between seaweed presence and species was not statistically clear. A greater sample size in the present study could have resolved this difference.

The effects of alternative resources were also different depending on the functional response parameter, having a significant effect on the handling time, but not the attack rate, in the present study. The attack rate corresponds to the search efficiency of consumers, reflecting the initial slope of functional response curves. Therefore, as this parameter reflects interaction strengths at low resource densities, the occurrence of alternative resources did not modulate feeding rates towards low‐density prey populations in any of the tested species here. Conversely, the handling time corresponds to effects at high prey densities, reciprocally determining the maximum feeding rate (i.e., curve asymptote). Significantly longer handling times from the native species in our study therefore indicate a greater amount of time required to physiologically process prey items, including consumption and digestion, in the presence of alternative seaweed resources, despite similar sizes among predators. However, it may also relate to the provisioning of shelter by seaweed disks, which may increase resting times over the experimental duration and lengthen handling times of predators. While the extent of feeding on seaweeds was not recorded formally here (although observed in pilot trials), our comparative results suggest that the native *G. locusta* has a high propensity to feed on *Fucus* sp. (or its epiphytes), even in the presence of abundant animal protein.

It is, however, noteworthy that the behavior of chironomid prey could further mediate these predator–prey interactions; we observed prey aggregation among seaweed disks, which might have affected predation efficiency. Indeed, benthic habitat structure is known to significantly mediate predator–prey dynamics (Barrios‐O'Neill et al., 2015), and this and other context‐dependencies such as temperature and salinity could be assessed by performing functional response experiments in the wild over different seasons and locations. Moreover, the two invasive alien species here may be more naïve to *Fucus* sp., with which the native *G. locusta* is commonly associated (Andersson et al., 2009). Foraging propensities may thus change over eco‐evolutionary timescales, resulting in an increased preference for this seaweed with increased exposure by invaders. Future studies should also consider preferences and switching propensities between multiple resource types at different available proportions. Given the present study considered single populations, which had been kept in the laboratory for an extended time, albeit under controlled conditions, consideration for interpopulation variability in impact should also be made in future studies. Particularly, as *P. maeoticus* was collected from its native range here, feeding rates from its invaded areas may differ, as has been shown in other trophic groups (e.g., Boets et al., 2019). In addition, given the reported effects of sex and reproductive status on gammarid functional responses (Dalal et al., 2020), future studies on these species should consider their effects on interaction strength, as we did not distinguish predator sex or reproductive status here.

Previous studies have already indicated a strong preference for animal prey by the widespread and ecologically impactful invader *G. tigrinus*, with an increasing feeding rate at higher temperatures also reported (Pellan et al., 2015). In our study, the predatory functional response of this species was least affected by alternative resources. With a broad salinity tolerance in its native range—including fully marine conditions—this species has had high invasion success in European fresh and brackish waters (Cuthbert et al., 2020), and is spreading rapidly through the Baltic Sea (Rewicz et al., 2019). The second invasive alien species in our study, *P. maeoticus*, has a more limited invasion history at present, including Ukrainian and Turkish waters (Alexandrov et al., 2007; Özbek, 2011). While not established yet in the Baltic Sea, a congeneric species, *Pontogammarus robustoides* Birstein 1932, is widely present in the system and known to be highly impactful (Grabowski et al., 2007); this species was, however, also unavailable in our region in the present study. In general, Ponto‐Caspian species are known to be disproportionately successful invaders due to high ecological plasticity associated with wide temperature and salinity tolerances (Cuthbert et al., 2020; Pauli et al., 2018). Here, we demonstrated that the predation rate of this Ponto‐Caspian invader was not significantly reduced by seaweed presence, although there was a slight tendency for reduced maximum feeding rates. We also note that the body size of this predator was generally larger than *G. locusta* and *G. tigrinus*, and thus their feeding rates may be conservative in comparison.

Overall, our results suggest that impact assessments for existing and emerging invasive alien species should increasingly consider biotic contexts, such as the simultaneous availability of multiple resources. Here, with a study system of three gammarids, our results support reduced interaction strengths in native *G. locusta* compared with the two invaders. Whereas the present study comprised a model system of three native or invasive alien gammarids, future studies should test alternative resource effects considering other taxonomic groups across a larger suite of species.

## AUTHOR CONTRIBUTIONS

**Ross N. Cuthbert:** Conceptualization (equal); formal analysis (equal); investigation (equal); methodology (equal); supervision (equal); visualization (equal); writing – original draft (equal). **Syrmalenia G. Kotronaki:** Conceptualization (equal); writing – review and editing (equal). **Jasmin C. Hütt:** Investigation (equal); writing – review and editing (equal). **Elisabeth Renk:** Investigation (equal); writing – review and editing (equal). **Niklas Warlo:** Investigation (equal); writing – review and editing (equal). **Elizabeta Briski:** Conceptualization (equal); methodology (equal); supervision (equal); writing – review and editing (equal).

## CONFLICT OF INTEREST

The submitted work was not carried out in the presence of any personal, professional, or financial relationships.

## Data Availability

Underlying data are available on Dryad (https://doi.org/10.5061/dryad.xsj3tx9jr).
